# A genetically modified *Plasmodium berghei* parasite as a surrogate for whole-sporozoite vaccination against *P. vivax* malaria

**DOI:** 10.1038/s41541-022-00585-8

**Published:** 2022-12-16

**Authors:** Diana Moita, Teresa G. Maia, Miguel Duarte, Carolina M. Andrade, Inês S. Albuquerque, Ankit Dwivedi, Joana C. Silva, Lilia González-Céron, Chris J. Janse, António M. Mendes, Miguel Prudêncio

**Affiliations:** 1 Instituto de Medicina Molecular João Lobo Antunes, Faculdade de Medicina da Universidade de Lisboa, Lisboa, Portugal; 2 Institute for Genome Sciences, University of Maryland School of Medicine, Baltimore, MD USA; 3 Centro Regional de Investigación en Salud Pública, Instituto Nacional de Salud Pública, Tapachula, Chiapas México; 4 Department of Parasitology, Leiden University Medical Center, Leiden, Netherlands

**Keywords:** Vaccines, Infectious diseases

## Abstract

Two malaria parasite species, *Plasmodium falciparum* (*Pf*) and *P. vivax* (*Pv*) are responsible for most of the disease burden caused by malaria. Vaccine development against this disease has focused mainly on *Pf*. Whole-sporozoite (WSp) vaccination, targeting pre-erythrocytic (PE) parasite stages, is a promising strategy for immunization against malaria and several *Pf*WSp-based vaccine candidates are currently undergoing clinical evaluation. In contrast, no WSp candidates have been developed for *Pv*, mainly due to constraints in the production of *Pv* sporozoites in the laboratory. Recently, we developed a novel approach for WSp vaccination against *Pf* based on the use of transgenic rodent *P. berghei* (*Pb*) sporozoites expressing immunogens of this human-infective parasite. We showed that this platform can be used to deliver PE *Pf* antigens, eliciting both targeted humoral responses and cross-species cellular immune responses against *Pf*. Here we explored this WSp platform for the delivery of *Pv* antigens. As the *Pv* circumsporozoite protein (CSP) is a leading vaccine candidate antigen, we generated a transgenic *Pb* parasite, *Pb*viVac, that, in addition to its endogenous *Pb*CSP, expresses *Pv*CSP under the control of a strictly PE promoter. Immunofluorescence microscopy analyses confirmed that both the *Pb*CSP and the *Pv*CSP antigens are expressed in *Pb*viVac sporozoites and liver stages and that *Pb*viVac sporozoite infectivity of hepatic cells is similar to that of its wild-type *Pb* counterpart. Immunization of mice with *Pb*viVac sporozoites elicits the production of anti-*Pv*CSP antibodies that efficiently recognize and bind to *Pv* sporozoites. Our results warrant further development and evaluation of *Pb*viVac as a surrogate for WSp vaccination against *Pv* malaria.

## Introduction

Malaria prevails as one of the deadliest infectious diseases worldwide, remaining a major public health concern, especially in the tropical and subtropical regions. In 2020 alone, the World Health Organization (WHO) estimated 241 million new clinical cases and 627,000 malaria-associated deaths, with the WHO African and Southeast Asian regions accounting for most global malaria cases^1^. In humans, malaria can be caused by several *Plasmodium* species, but *P. falciparum* (*Pf*) and *P. vivax* (*Pv*) are still responsible for most of the disease burden worldwide^2^. Although *Pf* remains the deadliest malaria parasite, *Pv* is the most geographically widespread^2^, and is increasingly recognized as a cause of severe disease^3^.

Mammalian infection by malaria parasites is initiated when an infected *Anopheles* mosquito deposits *Plasmodium* sporozoites, the parasite’s liver-infective forms, into the host’s skin and skin vasculature. Sporozoites then travel to the liver, where they infect hepatocytes and initiate an asymptomatic phase of asexual replication and parasite growth^4^. This process culminates in the formation of thousands of parasites, which are released into the bloodstream, where they invade, asexually replicate, egress, and reinvade host erythrocytes, in a continuous cycle that is responsible for malaria symptoms^5^. Importantly, unlike *Pf*, *Pv* parasites can generate dormant liver forms, termed hypnozoites, which may reactivate and lead to disease relapses long after the initial mosquito bite^6^.

Vaccines targeting the pre-erythrocytic (PE) stages of *Plasmodium* parasites, i.e., sporozoites and liver stages, constitute the most attractive approach to prevent malaria and are still the primary vaccination strategy to tackle *Pf* (reviewed in^7^). Recently, the WHO recommended the administration of the subunit vaccine RTS,S/AS01 (RTS,S) to children living in regions of moderate-to-high malaria transmission^8^. RTS,S specifically targets the *Pf* circumsporozoite protein (CSP), the most abundant antigen on the sporozoite surface. This vaccine demonstrated ~30% reduction in severe malaria cases in phase 3 clinical trials performed in African countries^9^. Nonetheless, RTS,S’s relatively low and short-lived efficacy^9^ underscores the need to develop vaccines with higher and more durable protection. An alternative to subunit vaccines is the use of whole-sporozoite (WSp) immunization strategies, based on the administration of live attenuated *Plasmodium* sporozoites to induce efficient immune responses against the PE parasite stages, precluding erythrocytic infection and, thus, clinical symptoms and further transmission. These include radiation-attenuated sporozoites^10^, genetically-attenuated parasites^11^ and immunization with fully infectious parasites under chemoprophylaxis^12^. Such systems have successfully been developed for *Pf* vaccination, with promising results in the clinic^13,14^. Conversely, the most advanced candidate for vaccination against *Pv* is still in early stages of clinical development^15^, and progress made in the development of *Pf* WSp vaccines is far from achieved for *Pv*. In fact, these vaccines depend on the availability of *Plasmodium* sporozoites, the liver-infective form of malaria parasites, and while *Pf* sporozoites can be easily obtained under laboratory conditions, it is currently impossible to successfully maintain in vitro blood stage cultures of *Pv* for long periods of time^16^. Since *Pv* sporozoites can only be obtained from mosquitoes fed on *Pv*-infected blood, their availability depends on blood samples collected from naturally infected patients^16^, curtailing the possibilities of developing *Pv* sporozoite-based vaccines. As such, attempts at developing WSp vaccines against *Pv* are scarce, and no *Pv* WSp candidates have been clinically evaluated, leaving this important gap largely unaddressed.

Recently, we developed an alternative WSp vaccination approach based on the use of genetically modified rodent *P. berghei* (*Pb*) sporozoites as a platform to elicit cross-species immune responses, as well as deliver antigens of human-infective parasites, eliciting specific immune responses against the latter^17,18^. We have shown that *Pb* parasites are inherently safe for human use, as they are unable to develop in human erythrocytes, in what would be the symptomatic stage of the parasite’s life cycle^17^. In a phase 1/2a clinical trial, *Pb*Vac, a *Pb* parasite engineered to express the *Pf*CSP, was shown to induce cross-species cellular immune responses and functional antibodies against *Pf*, leading to an estimated 95% reduction in the *Pf* liver load of immunized volunteers at the dose employed^19^.

The clinical validation of the *Pb*-based WSp immunization strategy warranted the exploitation of the *Pb* platform for the delivery of *Pv* antigens. Since the *Pv*CSP is a leading vaccine candidate (reviewed in^20^ and^21^) we now constructed *Pb*viVac, a genetically modified *Pb* parasite that expresses *Pv*CSP, to be employed as a surrogate for *Pv* WSp vaccination. Here, we describe the generation and pre-clinical characterization of *Pb*viVac, showing that it retains the mosquito and hepatic infectivity levels of the parental *Pb* line. We further demonstrate that immunization of rodents with *Pb*viVac elicits the production of antibodies that efficiently recognize and bind to *Pv* sporozoites, validating the novel parasite line as a tool to potentially be employed for immunization against *Pv* malaria.

## Results

### Amplification and sequence comparison of the *Pv*CSP gene

**Fig. 1:**
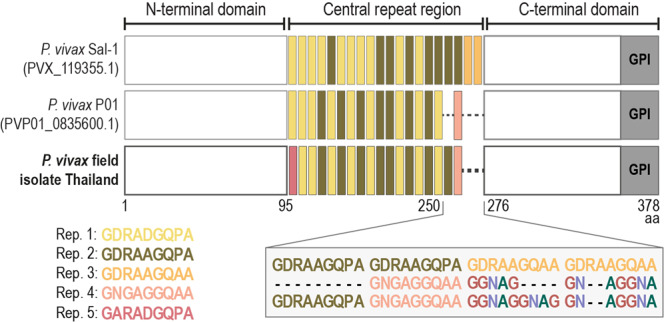
*Pv*CSP amino acid sequence used for generation of *Pb*viVac and comparison with reference *Pv* genomes. Comparison of the *Pv*CSP sequence used for generation of *Pb*viVac with those of reference *Pv* genomes. Schematic representation of the N-terminal domain, central repeat region and C-terminal region, with color blocks representing different repeat motifs (Rep.). Inset shows alignment of the region between amino acids (aa) 250 and 276.

In order to generate a transgenic *Pb* line that expresses a leading vaccine candidate antigen, *Pv*CSP, the *Pv*CSP coding sequence of a *Pv* field isolate from Thailand was initially amplified and compared to reference *Pv*CSP sequences, including those of *Pv* strains P01 and Sal-1 (Fig. 1 and Supplementary Fig. 1). As expected, the coding regions of both the N- and C-termini were highly conserved among the different *Pv*CSP sequences, with a single non-synonymous polymorphic site at position 38, resulting in a transition from an asparagine to a glycine in the *Pv* Thailand isolate (Supplementary Fig. 1), while most variability occurred in the protein’s central repeat region (Fig. 1). Our results indicate that the sequence of the *Pv*CSP gene present in the Thailand field isolate employed in our study is similar to that of the most common and well-adapted variant of the *Pv*CSP protein, VK210. This isolate presents 16 repeats of the VK210 variant’s most common peptide repeat motifs, GDRA(D/A)GQPA^22^, as well as a single occurrence of two other repeat motifs, GARADGQPA and GNGAGGQAA, the latter of which is also found in *Pv* strain Sal-1, as well as in Sri Lanka’s^23^ and Brazil’s^24^ *Pv* populations.

### Generation of the *Pb*viVac parasite line

A transgenic *Pb* line that expresses *Pv*CSP in addition to its endogenous *Pb*CSP, termed *Pb*viVac, was generated using the GIMO method of transfection^25^, as previously described for *Pb*Vac^17^ (Supplementary Fig. 2A). Briefly, the transgenic GIMO_*Pb*ANKA_ line (hereafter referred to as *Pb*WT), containing a positive-negative selection marker (*hdhfr::yfcu*) stably integrated into the silent *230p* locus, was transfected by double cross-over homologous recombination with a plasmid containing the *Pv*CSP-encoding gene fused to the *230p* targeting region. This resulted in the replacement of the selection marker by the *Pv*CSP gene and in its insertion into the *230p* locus of the *Pb* genome under the control of the 5’- and 3’-regulatory sequences of the *Pbuis4* gene, which is expressed exclusively in infective sporozoites and developing liver stages^26^. Following transfection, a clonal population was obtained by negative selection of a single transfected parasite employing the 5-fluorocytosine (5-FC) drug (Supplementary Fig. 2A). Clonal expansion resulted in two independent clones, *Pb*viVac #cl1 and *Pb*viVac #cl2, selected for further analysis. The correct integration of the *Pv*CSP expression cassette into the inert *230p* locus, as shown by the absence of the *hdhfr::yfcu* selection marker, and the correct integration of the construct into the genome, were confirmed through genotype analysis by diagnostic PCR analysis of both *Pb*viVac clones (Supplementary Fig. 2B).

### Production of *Pv*CSP-expressing sporozoites by *Pb*viVac

**Fig. 2:**
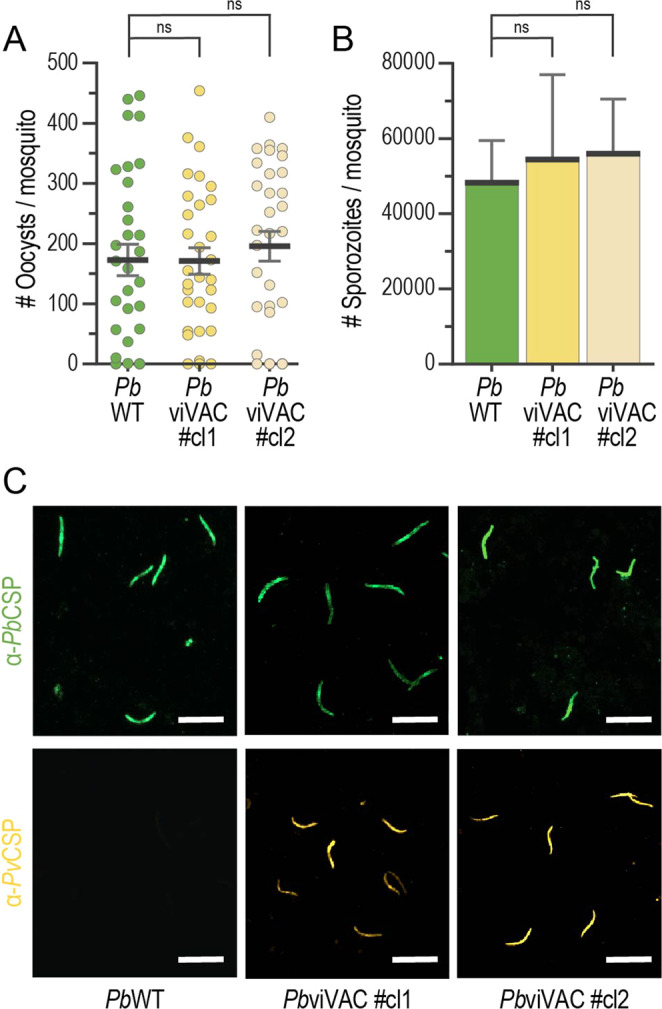
*Pb*viVac sporogonic development and *Pv*CSP expression by salivary gland sporozoites. **A, B** Midgut oocyst and salivary gland sporozoite numbers in *Pb*WT and *Pb*viVac-infected mosquitoes (*n* ≥ 28 mosquitoes per group); **C** Representative immunofluorescence microscopy images of *Pb*CSP (green) and *Pv*CSP (yellow) expressed by *Pb*WT (left) and *Pb*viVac (middle and right) sporozoites. Scale bar: 20 µm. Measurements were taken from distinct samples. The black lines/bars and grey lines correspond to mean and standard error of the mean, respectively (ns: not significant, Mann–Whitney *U* test).

Since relatively high numbers of sporozoites are required to elicit sterile immunity against malaria employing WSp vaccines^27^, we assessed the potential impact of the genetic manipulation of the *Pb* parasite on the sporogonic development of *Pb*viVac. To this end, midgut oocyst and salivary gland sporozoite numbers in mosquitoes fed on the blood of *Pb*viVac-infected mice were quantified 10 and 20–22 days after mosquito infection, respectively. Our results show that the *Pb*WT and *Pb*viVac parasite lines present comparable numbers of oocysts (Fig. 2A) and sporozoites (Fig. 2B) in the mosquito host’s midgut and salivary glands, respectively, indicating that the insertion of the *Pv*CSP gene in the *Pb* genome did not significantly impact the resulting transgenic parasite’s mosquito infectivity. We then analysed the expression of the endogenous *Pb*CSP and heterologous *Pv*CSP by *Pb*viVac sporozoites. Immunofluorescence microscopy analysis confirmed that while only *Pb*CSP is expressed by control *Pb*WT sporozoites, *Pb*viVac parasites express both *Pb*CSP and *Pv*CSP, as expected from the placement of the *Pv*CSP gene under the control of the PE *Pbuis4* promoter (Fig. 2C).

### In vitro and in vivo hepatic infection by *Pb*viVac parasites

**Fig. 3:**
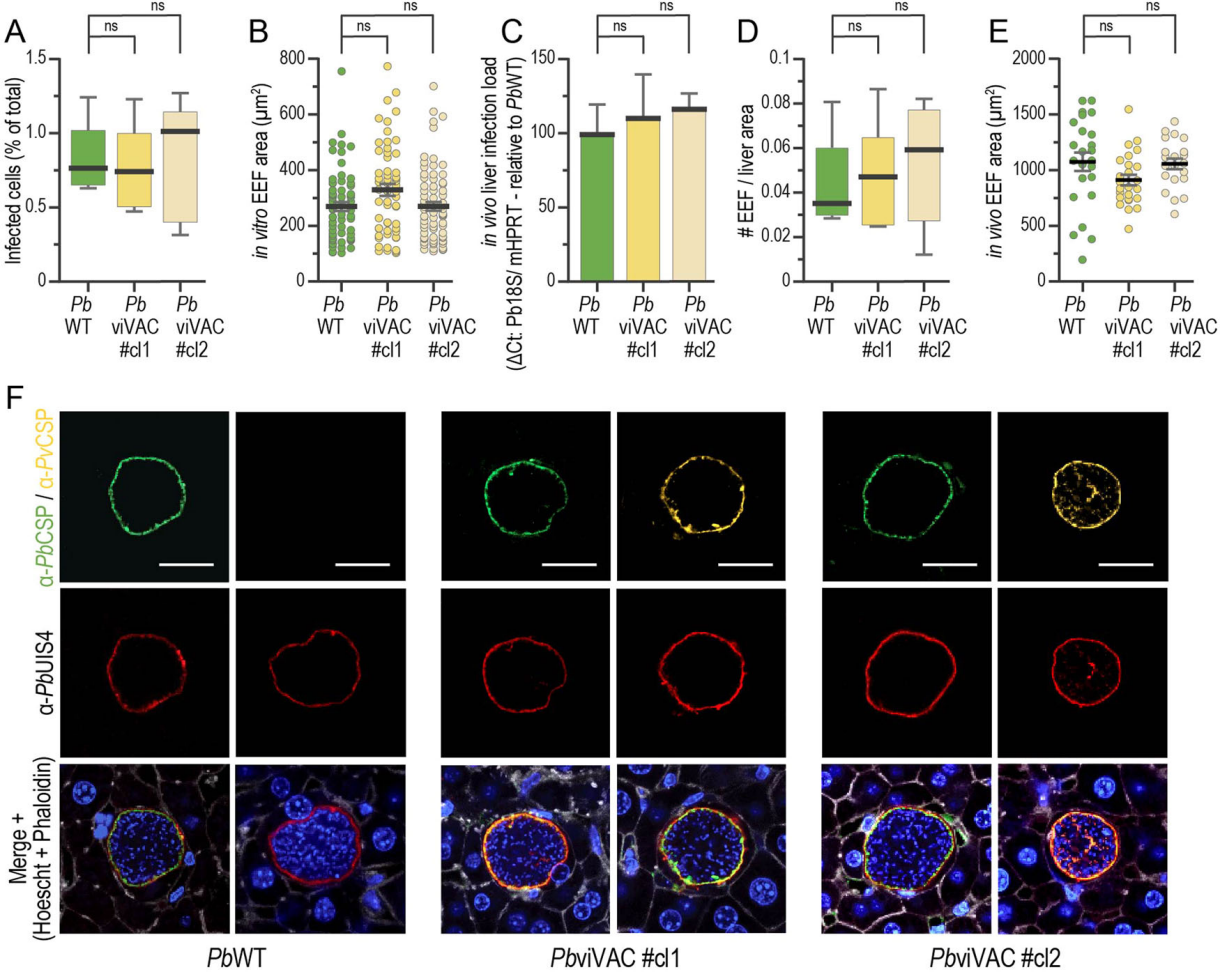
*Pb*viVac in vitro and in vivo pre-erythrocytic development and expression of *Pv*CSP. **A, B** Compared in vitro infectivity and parasite development of *Pb*WT and *Pb*viVac parasites in HepG2 human hepatoma cells (*n* ≥ 3 coverslips per group); Compared in vivo infectivity and development of *Pb*WT and *Pb*viVac parasites as determined by qPCR analysis of infected mouse livers **(C)**, quantification of the number of parasites per liver area **(D)**, and development of hepatic parasites **(E)** (*n* = 3 mice per group); **(F)** Representative immunofluorescence microscopy images of *Pb*WT and *Pb*viVac parasites developing in mouse livers 48 hpi. Immunofluorescence staining with the anti-*Pb*CSP (green) and anti-*Pv*CSP VK210 (yellow), as well as with anti-*Pb*UIS4 antibodies, confirms the expression of both proteins by *Pb*viVac and their localization to the parasite membrane. Scale bar: 20 µm. Measurements were taken from distinct samples. The boxes correspond to the 25th and 75th percentiles in (**A**) and (**D**) and the black lines/bars and grey lines correspond to mean and standard error of the mean, respectively (ns: not significant, Mann–Whitney *U* test).

Having shown that the sporogonic development of the *Pb*viVac parasite was not impaired by the transgenesis procedure employed in its generation, we then sought to evaluate the parasite’s hepatic infectivity in vitro employing the HepG2 and Huh7 human hepatoma cell lines. Immunofluorescence microscopy analysis (IFA) of infected HepG2 cells revealed that the infection rates (Fig. 3A) and hepatic parasite area at 48 h post-infection (hpi) (Fig. 3B) of both clones of the *Pb*viVac parasite are similar to those of *Pb*WT. Our data further showed that both *Pb*CSP and *Pv*CSP are expressed by developing hepatic *Pb*viVac parasites and are present at the parasite’s parasitophorous vacuole membrane (PVM), while, as expected, *Pb*WT parasites only express *Pb*CSP (Supplementary Fig. 3). Similar results were obtained following infection of Huh7 cells (Supplementary Fig. 2A–C).

We next sought to evaluate the liver infectivity of both parasite lines in vivo, employing the C57BL/6 J mouse model. Quantitative real-time PCR (qPCR) and IFA of livers from infected mice revealed similar overall infection loads for *Pb*viVac and *Pb*WT (Fig. 3C), with equivalent numbers of exoerythrocytic forms (EEFs) (Fig. 3D) and identical in vivo development (Fig. 3E). IFA of infected liver sections further showed that both *Pb*CSP and *Pv*CSP are expressed at the PVM during the liver stage of parasite development (Fig. 3F). Altogether, our analyses showed that *Pb*viVac parasites infect and develop inside hepatocytes similarly to the *Pb*WT control parasites, and that they express the heterologous *Pv*CSP throughout their PE development.

### Immunization of rodents with *Pb*viVac elicits *Pv* sporozoite-specific humoral immune responses

**Fig. 4:**
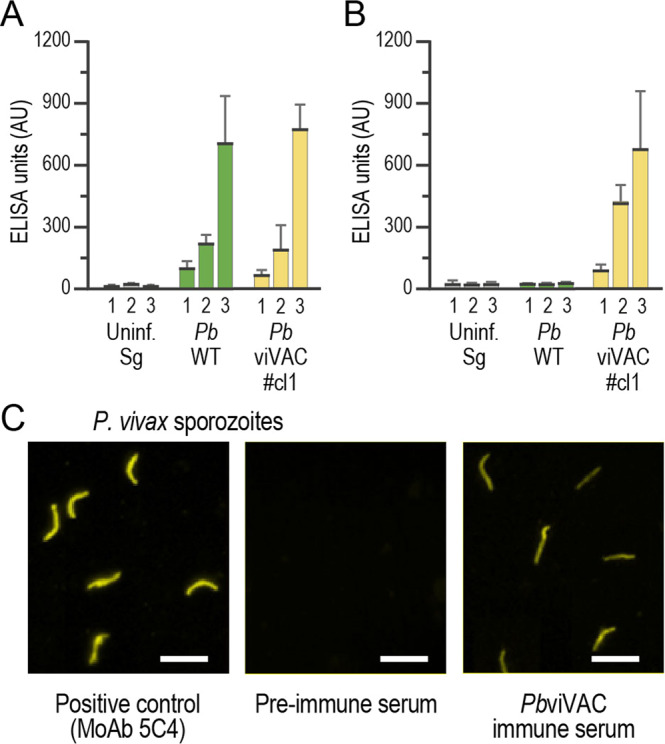
Humoral immune responses induced by immunization with *Pb*viVac. Total IgG titers against the *Pb*CSP **(A)** or the *Pv*CSP **(B)** repeat sequences in mouse plasma after prime (1), first (2) and second boost (3) immunization with either a mock control of uninfected mosquito salivary glands (grey), with *Pb*WT (green) or with *Pb*viVac (yellow) sporozoites; **(C)** Representative immunofluorescence microscopy images showing the binding of serum from *Pb*viVac-immunized mice to *Pv* sporozoites. AU: arbitrary units; Scale bar: 20 µm. The black lines/bars and grey lines correspond to mean and standard error of the mean, respectively.

Having confirmed the fitness of *Pb*viVac parasites throughout their life cycle, we then sought to evaluate their ability to elicit immune responses against human *Pv* parasites. To this end, C57BL/6 J mice were immunized by three intravenous injections of 1 × 10^4^ sporozoites of either *Pb*WT or *Pb*viVac under chloroquine coverage, with a one-week interval between immunizations. We started by analysing antigen-specific humoral immune responses of circulating IgGs in the plasma of immunized animals by enzyme-linked immunosorbent assay (ELISA), employing peptides spanning the repeat region of either *Pb*CSP (Fig. 4A) or of the VK210 variant’s *Pv*CSP (Fig. 4B). Our results show that the amount of anti-*Pb*CSP and anti-*Pv*CSP antibodies in the plasma of *Pb*viVac-immunized animals progressively increased after each of the three immunizations, whereas anti-*Pb*CSP, but no anti-*Pv*CSP antibodies, were detected in the plasma of the *Pb*WT-immunized mice.

We then investigated the ability of the antibodies elicited by immunization to recognize and bind to *Pv* sporozoites (Fig. 4C). IFA employing immune plasma collected before immunization with *Pb*viVac and one week after the final boost revealed antibody binding to immobilized native whole *Pv* sporozoites of the VK210 variant in the latter. Overall, our data show that immunization with *Pb*viVac sporozoites elicits a strong humoral response that efficiently recognizes and binds to *Pv* sporozoites.

### In silico analysis of predicted cross-species immunity between *Pb* and *Pv*

Our previous studies have indicated that rodent *Pb* parasites can elicit cross-species cellular immune responses against *Pf*, potentially resulting from the significant number of CD8^+^ T cell epitopes shared between the two species^17^. To assess the potential for cross-species cellular immunity between *Pb* and *Pv*, a comprehensive in silico analysis of the distribution of shared epitopes between both parasite species was performed (Supplementary Fig. 5). Our results indicate that, when analyses are limited to predicted CD8^+^ T cell epitopes with a top 0.5% binding affinity score, approximately 23.7 K distinct epitope sequences are shared between *Pb* and each of the *Pv* Sal-1 strains (reference sequence a from Salvador isolate). These epitope sequences map to 3376 independent *Plasmodium* orthologous proteins groups, 56.3% (3147/5585) of which are present in both species. Similarly, 23,829 predicted epitope sequences are shared between *Pb* and *Pv* P01, which map to 3379 ortholog groups, including 3139 that have homologs in both species. These numbers are comparable to those previously obtained from a comparison of the *Pb* and *Pf* 3D7 proteomes, where the 24,171 shared epitopes sequences mapped almost exclusively to 3223 orthologous protein pairs^17^. While further experimental evidence would be required to assess potential cross-species cellular immune responses between *Pb* and *Pv*, these data raise the possibility that, similarly to what was observed for *Pb*Vac and *Pf*^17,19^, immunization with *Pb*viVac may elicit some degree of T cell-mediated immunity against *Pv*.

## Discussion

Although the overall incidence of malaria cases associated with *Pf* has been declining outside Sub-Saharan, the prevalence of cases due to *Pv* is increasing, with this parasite species likely persisting as an obstacle to malaria eradication in the absence of an effective vaccine, which remains unavailable^28^. However, and in contrast to *Pf*, only very few *Pv* vaccine candidates have progressed to clinical evaluation. They include synthetic peptides, recombinant proteins and chimeric constructs comprising the N- and C-terminal regions as well as the central repeat region of the CSP (reviewed in^20^). One of the most advanced candidates, the Vivax malaria protein 1 (VMP001) vaccine, is a chimeric protein produced in *Escherichia coli* that comprises a truncated repeat region containing repeat sequences of the two most common *Pv* strains, VK210 and VK247^29^. In a phase 1/2a clinical trial, VMP001 demonstrated to be safe and induced strong humoral and CD4^+^ T cell immune responses to the vaccine antigen, resulting in a significant delay in patency^15^. More recently, a combination of three long synthetic peptides corresponding to the N-terminal, central repeats from the VK210 variant and C-terminal regions of the *Pv*CSP was evaluated in a phase 2a/2b clinical trial, revealing significant protection and immunogenicity in both naïve and semi-immune volunteers^30^.

Alternatively, WSp immunization approaches present a broader array of antigens to the immune system, potentially widening the range of immune responses elicited by vaccination. However, a *Pv*-based WSp vaccine remains unavailable, not least because the establishment of a robust system for in vitro culture of *Pv* has been hindered by several technical and logistical limitations that challenge not only vaccine production but also the future assessment of its efficacy in controlled human malaria infection (CHMI) trials^31^. Efforts to develop blood stage CHMI using *Pv* stabillates are currently ongoing and the blood of recipients can be used for mosquito feeds for *Pv* sporozoite generation. Nevertheless, although significant advances in the establishment of reproducible *Pv* CHMIs have been reported^32^, and the first trial to assess the efficacy of a *Pv*CSP vaccine candidate by CHMI was already successfully undertaken^15^, these are still dependent on the availability of blood from naturally infected patients to produce *Pv* sporozoites, and must take into account the need for elimination of hypnozoites.

Our study proposes an innovative approach to PE WSp vaccination against *Pv* that may help circumvent current limitations in production of *Pv* sporozoites in the laboratory^16^. The promising results obtained in a phase 1/2a clinical trial with the *Pb*Vac PE WSp vaccine candidate^19^, encouraged us to explore a similar strategy to deliver *Pv* antigens for immunization against this human-infective parasite. *Pb*viVac thus constitutes a potential surrogate for WSp immunization against *Pv* malaria, that is able to induce antibody immune responses against *Pv*CSP, as well as, potentially, cross-species cellular immunity targeting antigens conserved between the rodent and human malaria parasites^33,34^.

Protection induced by WSp vaccines targeting pre-erythrocytic stages has been reported to be mediated by both T cells and antibodies (reviewed in^33^). A possible advantage of the expression of *Pv* antigens by *Pb* relative to their incorporation in subunit vaccines is that a *Plasmodium*-based expression platform likely favours the correct folding of full-length *Pv* proteins, which may enhance the quality, quantity, and the repertoire of immune responses elicited by immunization^35,36^. Indeed, our results show that immunization of mice with *Pb*viVac elicits the production of anti-*Pv*CSP antibodies that efficiently recognize and bind to *Pv* sporozoites. This observation, alongside our ELISA data showing that these humoral responses include antibodies that specifically recognize the *Pv*CSP repeat region, suggest that they have the functional ability to inhibit hepatic infection by *Pv* sporozoites, similarly to what was observed for *Pb*Vac and *Pf*^17^. On the other hand, our in silico data reveal a high degree of CD8^+^ T cell epitope similarity between *Pb* and *Pv*, raising the possibility that immunization with *Pb*viVac might elicit some level of cellular immunity against *Pv*, as was observed for *Pb*Vac and *Pf*^17,19^. However, this possibility still requires experimental verification, which would add valuable information regarding the immunogenicity of this potential vaccine candidate and might constitute an important step on its path to the clinic.

*Pb*viVac expresses the *Pv*CSP from a Thailand field isolate, whose N- and C-terminal regions are highly conserved among multiple *Pv*CSP sequences, and are i) responsible for both CSP-specific and non-specific T cell and antibody responses^37–41^, and ii) contain the TSR motif and region I, which are critical for invasion and protein conformational changes throughout the parasite’s development^42^. Contrarily to *Pf*, the repeat region of the *Pv*CSP exhibits genetic heterology indicating that a vaccine targeting only one *Pv* strain could lead to strain-specific immune responses, leaving populations susceptible to infection with the other circulating variants^43,44^. Thus, although VK210 is the most common variant of *Pv*CSP^22^, the expression of only this sequence on the *Pb* platform may represent a limitation of *Pb*viVac. In fact, a recombinant protein including the repeats of both *Pv*CSP VK210 and VK247, as well as of *P. vivax*-like CSP, has recently been generated and shown to elicit the production of high titers of antibodies against each of the variants following immunization of a mouse model^45^. Accordingly, and since previous studies demonstrated the absence of significant cross-reactivity among the different *Pv*CSP alleles in animals immunized with individual recombinant proteins (VK210, VK247 and *P. vivax*-like)^46^, the *Pb* platform may in the future be engineered to simultaneously express of the most common alleles of CSP, potentially eliciting protective immune responses against a wider range of *Pv* strains.

Vaccination approaches based on *Pb* parasites are inherently safe and versatile, given this parasite’s high amenability to genetic modification^17,18^. Since the presence of several neutral loci in the *Pb* genome^47^ enables the insertion of *Pv* genes besides CSP, future candidates may be designed to express not only additional PE antigens, such as the thrombospondin-related anonymous protein (TRAP)^48^ but also candidate immunogens from different stages of this parasite’s life cycle, such as the blood stage Duffy binding protein (DBP)^49^ or the transmission stage Pvs25 protein^50^. Thus, our results establish not only a pre-clinical proof-of-concept for *Pb*-based vaccination against *Pv*, but also pave the way for the evaluation of *Pb*viVac or other candidates expressing additional *Pv* antigens in the clinic. Naturally, in order to be suitable for human vaccination, such candidates need to be produced under good manufacturing procedures (GMP) conditions. Since blood stage cultures of rodent malaria parasites have not yet been established, this could be achieved by feeding mosquitoes on infected Specific Pathogen Free rodents, followed by sporozoite purification and cryopreservation using the methods developed and established by Sanaria, Inc^51^.

In conclusion, our study shows for the first time that genetically engineered *Pb* parasites expressing *Pv* antigens may constitute a viable alternative to *Pv*-based WSp vaccines, overcoming the current limitations in producing GMP-compliant *Pv* sporozoites in the quantities required for vaccination^17^.

## Methods

### Animal experimental procedures

All animal experiments were performed at the animal Facility of Instituto de Medicina Molecular João Lobo Antunes. Male C57BL/6 J mice, aged six to eight weeks, were purchased from Charles River Laboratories (Lyon, France) and housed under specific pathogen-free (SPF) conditions. Experimental procedures were performed according to the Federation of European Laboratory Animal Science Associations (FELASA) guidelines and approved by IMM-JLA’s animal ethics committee (ORBEA-iMM). Mice were kept under a 12 h light/dark period at a temperature of 25 °C and 40–70% relative humidity. Filtered tap water and γ-irradiated pelleted diet were provided *ad libitum*.

### Parasite lines

The GIMO_*Pb*ANKA_ (henceforth referred to as *Pb*WT) mother line, which contains the human dihydrofolate reductase::yeast *cytosine deaminase* and *uridyl phosphoribosyl transferase* (*hdhfr::yfcu*) positive-negative selection markers in the silent *230p* locus, was employed to produce the two clonal lines of the transgenic *Pb*viVac parasite through the ‘gene insertion/marker out’ (GIMO) transfection method, as described below.

### Generation and genotyping of transgenic *P. berghei* parasite, *Pb*viVac

A transgenic *Pb* parasite line containing a *Pv*CSP expression cassette in the silent *230p* locus was generated using the GIMO technology^25^. A *Pv*CSP expression cassette was generated containing a *Pv*CSP coding sequence from a *Pv* isolate from Thailand and confirmed by sequencing (Supplementary Fig. 1; Stabvida sequencing services). The *Pv*CSP coding sequence is flanked by the 5′ and 3′ promoter and transcription terminator sequences of *Pbuis4*, which were amplified from wild-type *Pb* ANKA (*Pb*WT) genomic DNA. The GIMO technology was used to integrate integrates by double crossover homologous recombination into the neutral *230p* locus of the GIMO_*Pb*ANKA_ mother line, replacing the positive-negative selection marker *hdhfr::yfcu* cassette in the *Pb*WT mother line with the *Pv*CSP expression cassette (Supplementary Fig. 2). Transfected parasites were selected in vivo by applying negative selection by providing 5-fluorocytosine (5-FC) in the drinking water of mice. Selected transgenic parasites were cloned by the method of limiting dilution and two independent clones were selected for further characterization and analysis (*Pb*viVac #cl1 and *Pb*viVac #cl2). Correct integration of the construct into the genome of transgenic parasites was analysed by diagnostic PCR analysis of gDNA (Supplementary Fig. 2B) using the following primers sequences: p1654: GCAAAGTGAAGTTCAAATATG; p1494: AATTTAGTGGGATCCATATGC; p1901: GTTCGCTAAACTGCATCGTC; p1902: GTTTGAGGTAGCAAGTAGACG; p1497: TATAATTCATTATGAGTAGTGTAATTCAG; p1655: GAAATCGCAAACATAAGTATC.

### Mosquito infection and oocyst count

*Anopheles* (*A*.) *stephensi* mosquitoes were reared at iMM JLA-Lisboa at 27 °C and 80% humidity. Gametocyte-carrying infected mice were anesthetized and placed on top of a cage with previously starved female mosquitoes for about 30 min to allow mosquito biting. After the feeding, mosquitoes were incubated at 21 °C and 80% humidity and in a 12 h light/dark cycle. Ten days post infectious blood meal, mosquito midguts were hand-dissected and mounted in a glass microscope slide with 0.1% mercurochrome. Oocysts were then counted using an Olympus CKX41 inverted microscope.

### Sporozoite collection and imaging

Twenty to 22 days post infectious blood meal, sporozoites were obtained by dissection of salivary glands from infected female *A. stephensi* mosquitoes. Mosquito salivary glands were kept on ice in RPMI culture medium and homogenized with a pestle to release the sporozoites, which were subsequently counted on a Neubauer chamber using an Olympus CKX41 inverted microscope. For sporozoite imaging, 3 × 10^4^ sporozoites were placed on a 10-well slide (ThermoScientific Diagnosis Microscope slides) and left at room temperature (RT) until the wells were completely dry. Sporozoites were then fixed with 4% (v/v) paraformaldehyde (PFA; Santa Cruz Biotechnology) for 10 min and washed with PBS. Sporozoites were incubated with *Pb*-specific anti-*Pb*CSP (mAb 3D11), or *Pv*-specific anti-*Pv*CSP (mAb 2F2) antibodies in the presence of 0.25% (v/v) gelatine. The slides were left inside a humid chamber at 37 °C for 30 min. Following washing with PBS, slides were incubated with the secondary antibody anti-mouse Alexa-Fluor 488 (Jackson ImmunoResearch Laboratories) in the presence of Hoechst for 30 min at 37 °C. Each slide was then mounted with Fluoromount G (SouthernBiotech) and a cover slide. Confocal images were acquired using a Zeiss LSM 710 confocal microscope.

### In vitro infection of human hepatoma cell lines

Huh7 and HepG2 cells from human hepatoma cell lines were cultured in RPMI 1640 medium supplemented with 10% (v/v) fetal bovine serum (FBS), 1% (v/v) Penicillin/Streptomycin, 1% (v/v) Glutamine, 1% (v/v) non-essential amino acids and 1% (v/v) 4-(2-hydroxyethyl)-1-piperazine ethanesulphonic acid (HEPES), pH 7 and maintained at 37 °C with 5% CO_2_. For immunofluorescence microscopy analyses, cells were seeded (5 × 10^4^ per well) on glass coverslips in 24-well plates and infected 24 h later by adding 3 × 10^4^ freshly dissected sporozoites in supplemented RPMI containing Fungizone (1 μg/mL) and Gentamicin (50 μg/mL). Sporozoite addition was followed by centrifugation at 1800 x *g* for 5 min. Medium was replaced approximately 2 h post-infection (hpi) by fresh medium. Forty-eight hpi, cells were fixed with 4% (v/v) PFA for 20 min at RT and stored at 4 °C in PBS. Cells were incubated with the permeabilization/blocking solution (0.1% v/v Triton X-100, 1% w/v bovine serum albumin (BSA) in 1x PBS) for 30 min at RT. Parasites were stained with a *Pb*-specific goat anti-*Pb*UIS4 (1:450 dilution of a 2 mg/ml stock), *Pb*-specific mouse anti-*Pb*CSP (mAb 3D11; 1:200 dilution) or *Pv*-specific mouse anti-*Pv*CSP (mAb 2F2; 1:500 dilution) antibodies for 1 h at RT, followed by three washes with permeabilization/blocking solution. Cells were then incubated in a 1:300 dilution of anti-mouse Alexa-Fluor 488 (Jackson ImmunoResearch Laboratories) and anti-goat Alexa-Fluor 555 (Thermofisher) in the presence of 1:1000 dilution of Hoechst 33342 (Invitrogen) for nuclei staining. After 3 washes with PBS, coverslips were mounted in microscope slides with Fluoromount G (SouthernBiotech). Confocal images were acquired using a Zeiss LSM 710 confocal microscope. Widefield images for exoerythrocytic forms (EEFs) counting and parasite size determination were acquired on a Zeiss Axiovert 200 M widefield fluorescence microscope. Images were processed with ImageJ software (version 1.49b).

### In vivo infection of C57Bl/6 J mice and liver collection

C57BL/6 J mice were infected intravenously (i.v.), through retro-orbital injection of 3 × 10^4^ freshly collected sporozoites. Livers were collected at 44 hpi with the left lobes being snap-frozen in liquid nitrogen and stored at −80 °C for subsequent analysis; the remaining lobes were fixed on 4% PFA and stored at 4 °C for immunofluorescence microscopy analysis.

### RNA extraction, cDNA synthesis and qPCR analysis of hepatic infection

Liver lobes collected for qPCR analysis were homogenized in 3 mL of denaturing solution (4 M guanidine thiocyanate; 25 mM sodium citrate pH 7; 0.5% w/v *N*-lauroylsarcosine and 0.7% v/v β-mercaptoethanol in DEPC-treated water). Total RNA was extracted from liver homogenates using the Qiagen RNA extraction kit, according to the manufacturer’s instructions. The concentration of RNA in each sample was assessed by measurement of absorbance at 260 nm on a NanoDrop 2000 spectrophotometer. Complementary DNA (cDNA) was synthesized from 1 μg of RNA using the NZYTech First-Strand cDNA synthesis kit, according to the manufacturer’s instructions. The cDNA was synthesized in a Biometra Personal thermocycler employing the following parameters: 25 °C for 10 min, 55 °C for 30 min and 85 °C for 5 min. The qPCR reaction was performed in a total volume of 20 μL in an ABI Prism 7500 Fast system (Applied Biosystems) using the SYBR® Green Real-Time PCR Master Mix (BioRad). Parasite load was quantified using primers specific to *Pb* 18 S rRNA (forward/reverse: AAGCATTAAATAAAGCGAATACATCCTTAC/ GGAGATTGGTTTTGACGTTTATGTG). Mouse housekeeping gene hypoxanthine-guanine phosphoribosyltransferase (*Hprt*) expression was used for normalization (forward/reverse: TTTGCTGACCTGCTGGATTAC/ CAAGACATTCTTTCCAGTTAAAGTTG). Analysis of qPCR data was performed using the delta-delta relative quantification method.

### Immunofluorescence staining of liver sections

All experiments were performed at the Bioimaging Facility of Instituto de Medicina Molecular João Lobo Antunes. PFA-fixed liver lobes were cut into 50 μm sections using a vibratome (VT1000S, Leica) and were incubated in permeabilization/blocking solution (1% w/v BSA, 0.5% v/v Triton X-100 in PBS 1x) and IgG anti-mouse (1:150) at RT overnight. After three washes with PBS, liver sections were incubated for 2 h with a *Pb*-specific goat anti-*Pb*UIS4 (1:450 dilution of a 2 mg/ml stock) and mouse anti-*Pb*CSP (mAb 3D11; 1:200 dilution) or mouse anti-*Pv*CSP (mAb 2F2; 1:500 dilution) antibodies. Following primary antibody incubation, sections were washed thrice with PBS 1x and incubated with the following secondary antibodies: 1:500 dilution of anti-mouse Alexa-Fluor 488 (Jackson ImmunoResearch Laboratories), 1:500 dilution of anti-goat Alexa-Fluor 555 (Thermofisher) and 1:50 dilution of Alexa-Fluor 660 Phalloidin (Thermofisher) for actin staining in the presence of 1:150 dilution of Hoechst 33342 (Invitrogen). After washing, the liver sections were mounted on microscope slides with Fluoromount G (SouthernBiotech). Widefield images for hepatic infection and parasite size determination were acquired in a Zeiss Axiovert 200 M microscope. Confocal images were acquired using a Zeiss LSM 710 confocal microscope. Images were processed with ImageJ software (version 1.49b).

### Immunization of C57BL/6 J mice

In order to analyse the humoral responses elicited by *Pb*viVac parasites, C57BL/6 J mice were immunized i.v., through three intravenous retro-orbital injections of 1 × 10^4^ freshly collected sporozoites from either *Pb*WT or *Pb*viVac parasite lines or with an extract obtained from the dissection of an identical number of non-infected mosquito salivary glands, with one-week intervals between immunizations and daily administration of chloroquine (35 mg/kg/mouse weight) to prevent the establishment of blood stage infection. Before each immunization and one week after the final one, blood was collected and centrifuged at 10,000 *x* *g* for 10 min to separate the red blood cells from the plasma. Plasma was then stored at −80 °C until further analysis either by ELISA or IFA.

### ELISA for anti-*Pb*CSP and anti-*Pv*CSP antibodies

High protein-binding capacity 96-well enzyme-linked immunosorbent assay (ELISA) plates (Nunc MaxiSorp^TM^ flat-bottom) were coated with synthetic peptide (Sigma) based on the VK210 variant repeat region of *Pv*CSP with the amino acid sequence GD RAD GQP AGD RAA GQP A, or the repeat region of the *Pb*CSP with the amino acid sequence (CPPPPNPN)2. The peptide was coated overnight at 4 °C at a concentration of 5 μg/ml in a volume of 50 μl per well. Plates were washed three times with PBS containing 0.1% (v/v) Tween-20 and blocked with 200 μl PBS containing 0.1% (v/v) Tween-20 and 1% (w/v) BSA for 30 min at RT. Plates were washed one additional time and samples serially diluted in PBS containing 0.1% (v/v) Tween-20 and 1% (w/v) BSA were added and incubated at 22 °C for 2 h. After washing four times, horseradish peroxidase-labelled goat anti-mouse IgG (GE Healthcare UK) was added at a dilution of 1:2000 and incubated at 22 °C for 1 h. BD OptEIA^TM^ TMBnSubstrate Reagent was then added for development and incubated for 1 to 3 min at 22 °C before stopping the reaction by adding 50 μl Stop solution (2NH_2_SO_4_). The optical density was determined using a microplate reader (Infinite M200). To serve as a positive control and to allow comparison between samples from different assays, a standard titration curve of at least 8 points, starting a dilution of 1/20 of a pool of mouse plasma from all immunized animals, was used as reference in all assays.

### Immunofluorescence analysis on immobilized *Pv* sporozoites

*Pv* salivary gland sporozoites were obtained from infected *Anopheles albimanus* mosquitoes, 12–14 days after artificial blood feeding through parafilm membranes on *Pv*-infected blood^52,53^. About 1000 sporozoites were added per well in 8-well slides, air-dried, and preserved at −80 °C until used. IFA slides containing *Pv* VK210 sporozoites were thawed and air-dried. Blocking was done with PBS containing 3% (w/v) BSA. After washing with PBS containing 0.5% (w/v) BSA, mouse plasma was added in serial dilutions ranging from 1:40 to 1:400 overnight at 4 °C, followed by extensive washing with PBS containing 0.05% (w/v) Tween-20. FITC anti-mouse IgG+M (1:500; Invitrogen) was used as secondary fluorescent antibody and incubated for 1 h at RT. After washing three times with PBS containing 0.05% (w/v) Tween 20, slides were mounted in buffered glycerine and analysed by fluorescence microscopy using a Zeiss UV microscope. PBS was used as a negative control and two monoclonal antibodies against native *Pv*CSP repeats were used as positive controls in every slide. The negative control did not deliver any IFA signal detectable by visual examination, even at the lowest dilution.

### In silico identification of CD8^+^ T cell epitopes in the *Pv* and *Pb* proteomes

CD8^+^ T cell epitopes were predicted in the proteomes of *P. berghei* ANKARA (5,076 proteins) and *P. vivax* strains Sal 1 (5585 proteins) and P01 (6677 proteins) using the in silico epitope predictor netMHCpan (v4.0)^54^, as previously described^17^. Briefly, HLA types were chosen to represent 10 of the most frequent HLA-A and B supertypes, based on allele frequencies taken from the Allele Frequency Net Database^55^. Peptide lengths of 9,10, and 11 were used to search for 9-mer core epitopes. Strong binders, defined as peptides that are in the top 0.5% of binding affinity prediction scores, are reported here.

### Statistical analyses

Statistical analyses were performed using the GraphPad Prism 5 software. Results are expressed by mean ± SEM and statistical analyses were performed using the Mann-Whitney non-parametric test.

### Reporting summary

Further information on research design is available in the Nature Research Reporting Summary linked to this article.

## Supplementary information

Supplementary Material

REPORTING SUMMARY

## Data Availability

All data needed to evaluate the conclusions in this paper are present in the paper or the Supplementary Materials.
