# Supplementary material for: A genetically modified Plasmodium berghei parasite as a surrogate for whole-sporozoite vaccination against P. vivax malaria
Source: NPJ Vaccines. 2022 Dec 16;7:163. doi: 10.1038/s41541-022-00585-8 (PMC9755804; doi:10.1038/s41541-022-00585-8)
Supplement: Supplementary file 1 — Supplementary Material [file 41541_2022_585_MOESM1_ESM.pdf]

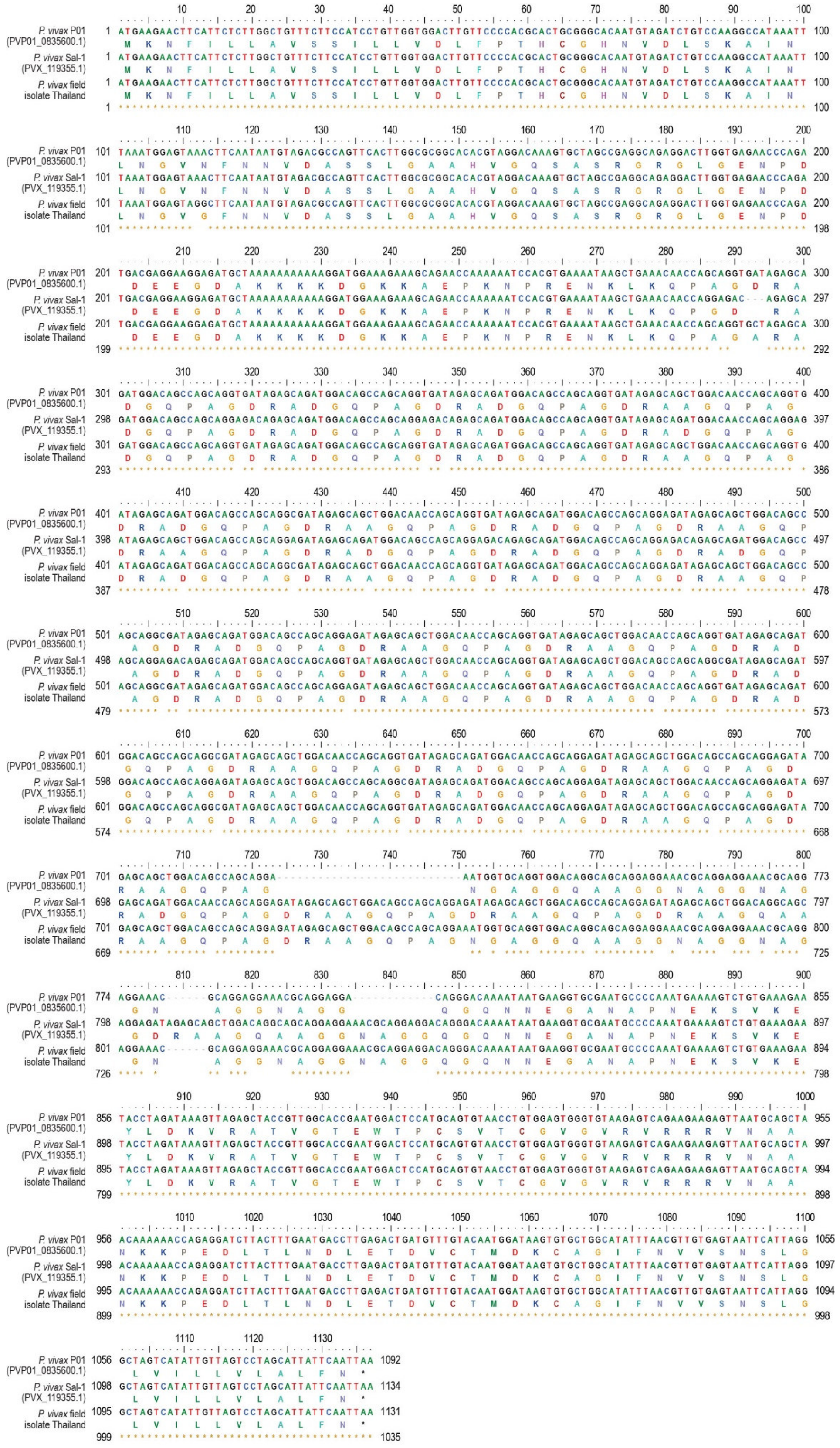

Supplementary Figure 1 - ClustalW alignment of the *Pv*CSP gene sequence used to generate *Pb*viVac with sequences from reference *Pv* genomes.

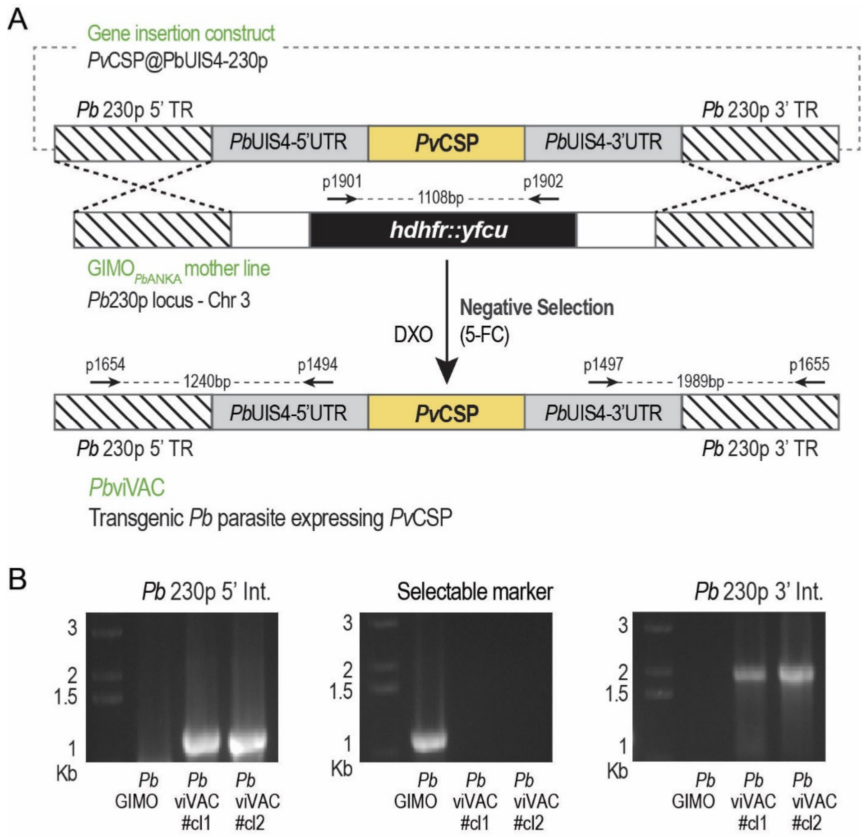

Supplementary Figure 2 - Generation and genotyping of the transgenic *Pb*viVac parasite line.

(A) Schematic representation of the strategy used to generate the transgenic *Pb*viVac line. The selectable marker (SM; *hdhfr*::*yfcu*) in the GIMO$_{Pb\mathrm{ANKA}}$ mother line was replaced by the *Pv*CSP coding sequence placed under the control of the *Pb*UIS4 gene promoter (5'-UTR) and *Pbuis4* transcriptional terminator sequence (3'- UTR) by double cross-over homologous recombination (DXO) using 5' and 3' targeting sequences (TR) for the neutral *230p* locus in chromosome 3. Subsequent administration of 5-fluorocytosine (5-FC) allows negative selection of SM-free parasites. Black arrows indicate the location and identifier of primers used for diagnostic PCR, as well as the expected PCR product sizes; **(B)** Genotype analysis by diagnostic PCR analysis of cloned parasite *Pb*viVac lines confirms correct integration of the *Pv*CSP expression cassette in the neutral *230p* locus, including correct integration at the 5' and 3'regions (5'int. and 3'int.), as well as the absence of selectable marker.

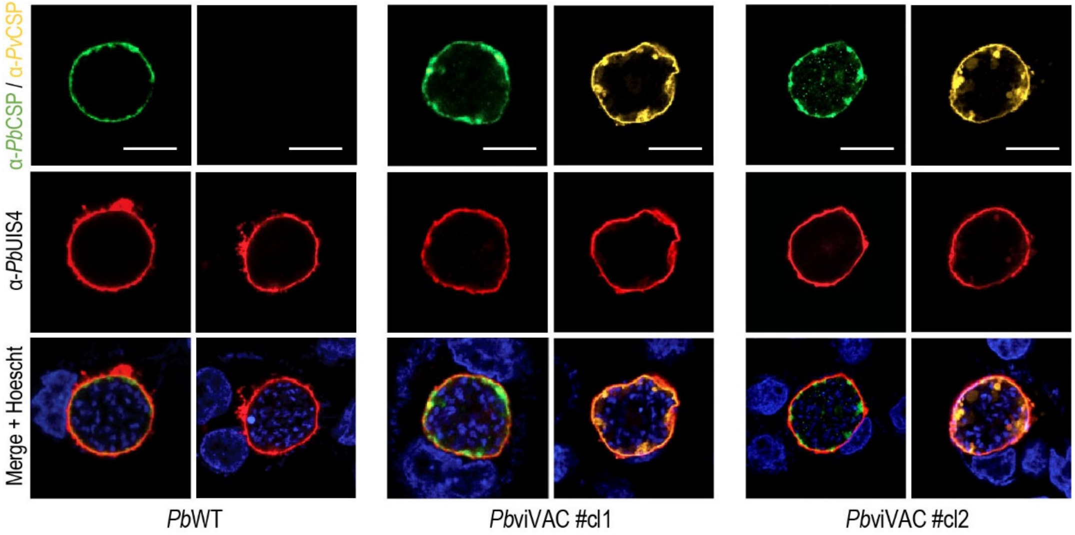

Supplementary Figure 3 – Expression of *Pv*CSP by *Pb*viVac parasites developing *in vitro* in HepG2 cells.

Representative immunofluorescence microscopy images of *Pb*WT and *Pb*viVac parasites developing in HepG2 hepatoma cells 48 hpi. Immunofluorescence staining with the anti-*Pb*CSP (green) and anti-*Pv*CSP VK210 (yellow), as well as with the anti-*Pb*UIS4 antibodies, confirms the expression of both proteins by *Pb*viVac and their localization to the parasite membrane. Scale bar: 20 µm.

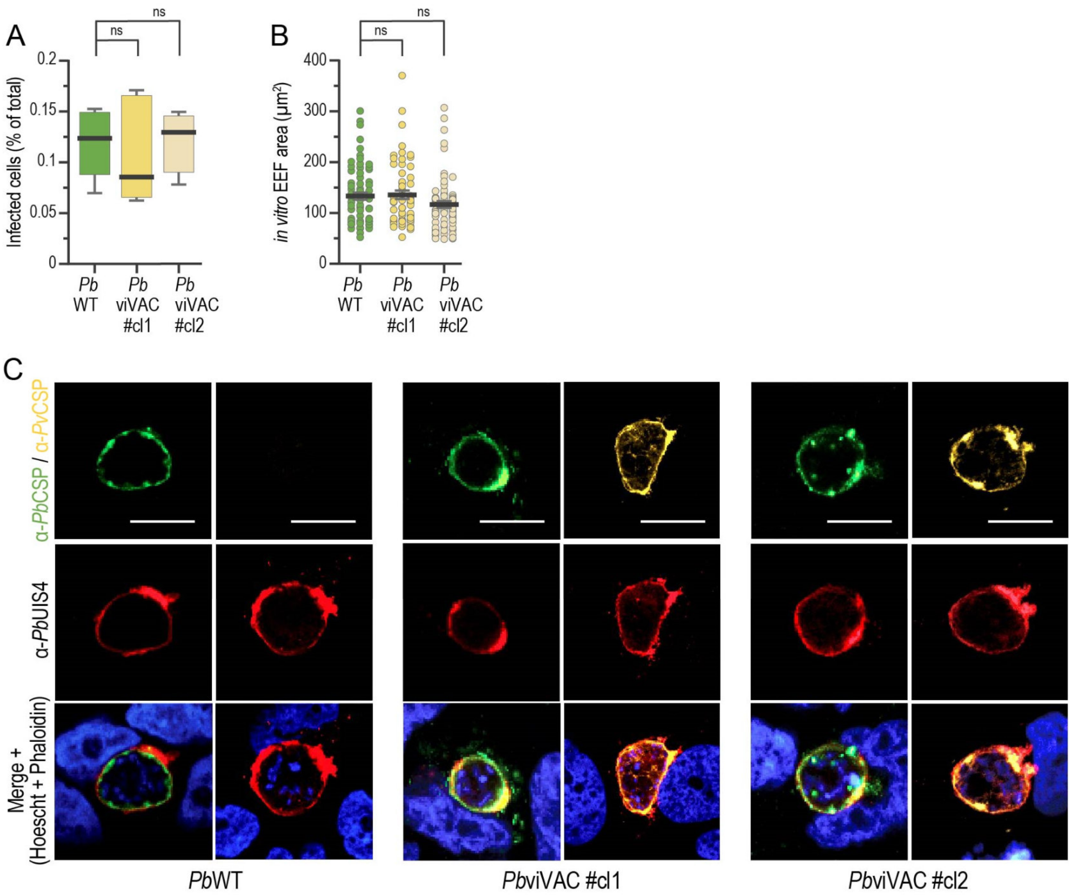

Supplementary Figure 4 - *Pb*viVac *in vitro* pre-erythrocytic development in Huh 7 human hepatoma cells and expression of *Pv*CSP.

(A,B) Compared *in vitro* infectivity **(A)** and parasite development **(B)** of *Pb*WT and *Pb*viVac parasites in Huh7 human hepatoma cells; **(C)** Representative immunofluorescence microscopy images of *Pb*WT and *Pb*viVac parasites developing in Huh7 cells at 48 hpi. Immunofluorescence staining with the anti-*Pb*CSP (green) and anti-*Pv*CSP VK210 (yellow), as well as anti-*Pb*UIS4 antibodies confirms the expression of both proteins by *Pb*viVac and their localization to the parasite membrane. Scale bar: 20 µm. The boxes correspond to the 25th and 75th percentiles in (A) and the black and grey lines correspond to mean and standard error of the mean, respectively (ns: not significant, Mann-Whitney *U* test).

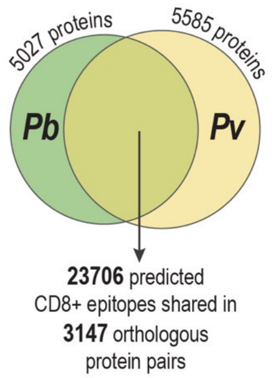

Supplementary Figure 5 - *In silico* analyses of *Pb* and *Pv* predicted $CD8^+$ T cell epitopes.

Distribution of orthologous protein pairs and strong binding epitopes shared between the *Pb* and *Pv* proteomes.
