# Supplementary material for: A genetically modified Plasmodium berghei parasite as a surrogate for whole-sporozoite vaccination against P. vivax malaria
Source: NPJ Vaccines. 2022 Dec 16;7:163. doi: 10.1038/s41541-022-00585-8 (PMC9755804; doi:10.1038/s41541-022-00585-8)
Supplement: Supplementary file 2 — REPORTING SUMMARY [file 41541_2022_585_MOESM2_ESM.pdf]

nature portfolio

Corresponding author(s): Dr Miguel Prudêncio

Last updated by author(s): Nov 9, 2022

Reporting Summary

Nature Portfolio wishes to improve the reproducibility of the work that we publish. This form provides structure for consistency and transparency in reporting. For further information on Nature Portfolio policies, see our Editorial Policies and the Editorial Policy Checklist.

Statistics

For all statistical analyses, confirm that the following items are present in the figure legend, table legend, main text, or Methods section.

n/a	Confirmed	
☐	☒	The exact sample size (*n*) for each experimental group/condition, given as a discrete number and unit of measurement
☐	☒	A statement on whether measurements were taken from distinct samples or whether the same sample was measured repeatedly
☐	☒	The statistical test(s) used AND whether they are one- or two-sided<br>*Only common tests should be described solely by name; describe more complex techniques in the Methods section.*
☒	☐	A description of all covariates tested
☒	☐	A description of any assumptions or corrections, such as tests of normality and adjustment for multiple comparisons
☐	☒	A full description of the statistical parameters including central tendency (e.g. means) or other basic estimates (e.g. regression coefficient) AND variation (e.g. standard deviation) or associated estimates of uncertainty (e.g. confidence intervals)
☒	☐	For null hypothesis testing, the test statistic (e.g. *F*, *t*, *r*) with confidence intervals, effect sizes, degrees of freedom and *P* value noted<br>*Give P values as exact values whenever suitable.*
☒	☐	For Bayesian analysis, information on the choice of priors and Markov chain Monte Carlo settings
☒	☐	For hierarchical and complex designs, identification of the appropriate level for tests and full reporting of outcomes
☒	☐	Estimates of effect sizes (e.g. Cohen's *d*, Pearson's *r*), indicating how they were calculated

Our web collection on statistics for biologists contains articles on many of the points above.

Software and code

Policy information about availability of computer code

Data collection	The complete proteomes of Pf and Pb were downloaded from PlasmoDB (v36). Allele frequencies of the alleles representative of the HLA-A and -B supertypes, were as described in the Allele Frequency Net Database. The proteins containing shared epitopes were compared to the file of orthologs obtained from Orthomcl.org (v5) (orthomcl.org/common/downloads/release-5/pairs/orthologs.txt.gz).
Data analysis	CD8+ T cell epitope prediction was performed with the package NetMHCpan (v4.0). The output of NetMHCpan (v4.0) was parsed to obtain all unique epitopes predicted across all HLA supertypes and across all epitope lengths. The proteins containing shared epitopes were compared to the file of orthologs obtained from Orthomcl.org (v5) (orthomcl.org/common/downloads/release-5/pairs/orthologs.txt.gz), to identify pairs of proteins belonging to the same orthologous group.

For manuscripts utilizing custom algorithms or software that are central to the research but not yet described in published literature, software must be made available to editors and reviewers. We strongly encourage code deposition in a community repository (e.g. GitHub). See the Nature Portfolio guidelines for submitting code & software for further information.

Data

Policy information about availability of data

All manuscripts must include a data availability statement. This statement should provide the following information, where applicable:

- Accession codes, unique identifiers, or web links for publicly available datasets
- A description of any restrictions on data availability
- For clinical datasets or third party data, please ensure that the statement adheres to our policy

Figure S5 was generated from analyses conducted on the output of NetMHCpan (v4.0), as described in Methods and in the Supplemental Information. The source of all datasets, as well as the software used, are detailed in Methods. The comprehensive list of unique predicted epitopes and proteins in which they fall are available from the corresponding author upon reasonable request.

Human research participants

Policy information about studies involving human research participants and Sex and Gender in Research.

Reporting on sex and gender	N/A
Population characteristics	N/A
Recruitment	N/A
Ethics oversight	N/A

Note that full information on the approval of the study protocol must also be provided in the manuscript.

Field-specific reporting

Please select the one below that is the best fit for your research. If you are not sure, read the appropriate sections before making your selection.

☒ Life sciences ☐ Behavioural & social sciences ☐ Ecological, evolutionary & environmental sciences

For a reference copy of the document with all sections, see nature.com/documents/nr-reporting-summary-flat.pdf

Life sciences study design

All studies must disclose on these points even when the disclosure is negative.

Sample size	No sample-size calculations were performed. For in vitro experiments employing cultured cells, and for in vivo experiments with mouse models, sample sizes ensuring statistical significance were employed.
Data exclusions	No data were excluded form the analyses.
Replication	All attempts at replication were successful.
Randomization	Experimental animals were age- and sex-matched, and were randomly distributed by experimental groups.
Blinding	No blinding was performed, as blinding was not required for the type of analyses performed. All the experimental parameters measured and analyzed were deemed objective and not subject to any bias introduced by the researchers.

Reporting for specific materials, systems and methods

We require information from authors about some types of materials, experimental systems and methods used in many studies. Here, indicate whether each material, system or method listed is relevant to your study. If you are not sure if a list item applies to your research, read the appropriate section before selecting a response.

Materials & experimental systems

n/a	Involved in the study	
☐	☒	Antibodies
☐	☒	Eukaryotic cell lines
☒	☐	Palaeontology and archaeology
☐	☒	Animals and other organisms
☒	☐	Clinical data
☒	☐	Dual use research of concern

Methods

n/a	Involved in the study	
☒	☐	ChIP-seq
☒	☐	Flow cytometry
☒	☐	MRI-based neuroimaging

Antibodies

Antibodies used	The study included the following antibodies:<br>- goat anti-P. berghei UIS4 (LifeSpan BioSciences, Inc. - LS-C204260)<br>- mouse anti-P. berghei CS. Monoclonal antibody produced in-house from the 3D11 hybridoma (BEI Resources MRA-100)<br>- mouse anti-P. vivax CS. Monoclonal antibody produced in-house from the 2F2 hybridoma (BEI Resources MRA-184)<br>- anti-mouse Alexa Fluor 488 (Jackson ImmunoResearch Laboratories, Code: 715-545-150)<br>- anti-goat Alexa Fluor 555 (ThermoFisher, catalog # A-21432)<br>- Alexa Fluor 660 Phalloidin (ThermoFisher, catalog # A-22285)<br>- Horseradish peroxidase-labeled goat anti-mouse IgG (GE Healthcare UK)<br>- FITC anti-mouse IgG+M (Invitrogen)
Validation	The anti-Plasmodium berghei UIS4 Antibody LS-C204260 has been validated by the manufacturer (LifeSpan BioSciences), as described in https://www.lsbio.com/antibodies/anti-plasmodium-berghei-uis4-antibody-if-immunofluorescence-ls-c204260/212516<br>The mouse anti-P. berghei CS. is a monoclonal antibody produced from the 3D11 hybridoma, as validated by BEI Resources (https://www.beiresources.org/Catalog/cellBanks/MRA-100.aspx)<br>The mouse anti-P. vivax CS. is a monoclonal antibody produced from the 2F2 hybridoma, as validated by BEI Resources (https://www.beiresources.org/Catalog/cellBanks/MRA-184.aspx)

Eukaryotic cell lines

Policy information about cell lines and Sex and Gender in Research

Cell line source(s)	HuH-7 is a well differentiated hepatocyte derived cellular carcinoma cell line deposited at the Japanese Collection of Research Bioresources under JCRB0403. The HepG2 cell line is a human hepatoma cell line deposited at ATCC under HB-8065.
Authentication	None of the cell lines were specifically authenticated for this study.
Mycoplasma contamination	All cell lines tested negative for mycoplasma contamination.
Commonly misidentified lines (See ICLAC register)	No commonly misidentified cell lines were used in the study.

Animals and other research organisms

Policy information about studies involving animals; ARRIVE guidelines recommended for reporting animal research, and Sex and Gender in Research

Laboratory animals	The study involved male wild-type C57BL/6 mice (Mus musculus) aged 6-8 weeks produced by Charles River France.
Wild animals	The study did not involve wild animals.
Reporting on sex	Findings of this study only apply to one sex, as gender is not expected to play a significant role in the infection and immune analyses performed.
Field-collected samples	The study did not involve field-collected samples.
Ethics oversight	Instituto de Medicina Molecular - João Lobo Antunes' animal ethics committee Órgão Responsável pelo Bem-Estar Animal (ORBEA-iMM)

Note that full information on the approval of the study protocol must also be provided in the manuscript.
